# 2-(3,4-Dichloro­phen­yl)-*N*-(1,5-dimethyl-3-oxo-2-phenyl-2,3-dihydro-1*H*-pyrazol-4-yl)acetamide

**DOI:** 10.1107/S1600536813002341

**Published:** 2013-02-20

**Authors:** Aneeka Mahan, Ray J. Butcher, Prakash S. Nayak, B. Narayana, H. S. Yathirajan

**Affiliations:** a Lake Braddock Secondary School, 9200 Burke Lake Road, Burke, VA 22015, USA; b Department of Chemistry, Howard University, 525 College Street NW, Washington DC 20059, USA; c Department of Studies in Chemistry, Mangalore University, Mangalagangotri 574 199, India; d Department of Studies in Chemistry, University of Mysore, Manasagangotri, Mysore 570 006, India

## Abstract

In the title compound, C_19_H_17_Cl_2_N_3_O_2_, there are three mol­ecules (*A*, *B* and *C*) in the asymmetric unit and each differs in the conformation adopted. As a result of steric repulsion, the amide group is rotated with respect to both the dichloro­phenyl and 2,3-dihydro-1*H*-pyrazol-4-yl rings, making dihedral angles of 44.5 (2) and 56.2 (2)°, respectively in *A*, 51.1 (2) and 54.1 (2)° in *B*, and 53.8 (2) and 54.6 (2)° in *C*. The dihedral angles between the dichloro­phenyl and 2,3-dihydro-1*H*-pyrazol-4-yl rings are 54.8 (2), 76.2 (2) and 77.5 (2)° in mol­ecules *A*, *B* and *C*, respectively, while the 2,3-dihydro-1*H*-pyrazol-4-yl and phenyl rings make dihedral angles of 45.3 (2), 51.2 (2) and 42.8 (2)°, respectively. In the crystal, two of the mol­ecules are linked through N—H⋯O hydrogen bonding to an adjoining mol­ecule, forming dimers of the *R*_2_^2^(10) type, while the third mol­ecule forms such dimers with itself. C—H⋯O inter­actions link the dimers.

## Related literature

For graph-set description of hydrogen-bonding patterns, see: Bernstein *et al.* (1995 ▶). For related structures, see: Fun *et al.* (2011*a* ▶,*b* ▶, 2012*a* ▶,*b* ▶). For similar structures but with differing dichloro substitution, see: Butcher *et al.* (2013*a* ▶,*b* ▶). For a description of the Cambridge Structural Database, see: Allen (2002 ▶). For the biological activity of *N*-substituted 2-aryl­acetamides, see: Mijin & Marinkovic (2006 ▶); Mijin *et al.* (2008 ▶). For the coordination abilities of amides, see: Wu *et al.* (2008 ▶, 2010 ▶).
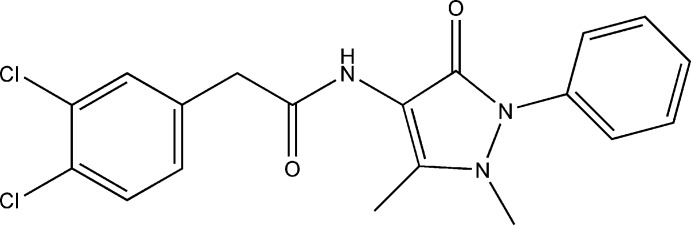


## Experimental

### 

#### Crystal data

C_19_H_17_Cl_2_N_3_O_2_
*M* *_r_* = 390.26
Monoclinic, 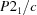

*a* = 17.2064 (8) Å
*b* = 20.7984 (9) Å
*c* = 15.6102 (7) Å
β = 101.213 (4)°
*V* = 5479.7 (4) Å^3^
*Z* = 12
Mo *K*α radiation
μ = 0.37 mm^−1^
*T* = 123 K
0.51 × 0.34 × 0.10 mm

#### Data collection

Agilent Xcalibur (Ruby, Gemini) diffractometer
Absorption correction: analytical [*CrysAlis PRO* (Agilent, 2011 ▶) based on expressions derived by Clark & Reid (1995 ▶)] *T*_min_ = 0.743, *T*_max_ = 0.932
54403 measured reflections
27521 independent reflections
11938 reflections with *I* > 2σ(*I*)
*R*_int_ = 0.076

#### Refinement

*R*[*F* ^2^ > 2σ(*F* ^2^)] = 0.119
*wR*(*F* ^2^) = 0.345
*S* = 1.02
27521 reflections
709 parameters
H-atom parameters constrained
Δρ_max_ = 3.46 e Å^−3^
Δρ_min_ = −0.84 e Å^−3^

### 

Data collection: *CrysAlis PRO* (Agilent, 2011 ▶); cell refinement: *CrysAlis PRO*; data reduction: *CrysAlis PRO*; program(s) used to solve structure: *SHELXS97* (Sheldrick, 2008 ▶); program(s) used to refine structure: *SHELXL97* (Sheldrick, 2008 ▶); molecular graphics: *SHELXTL* (Sheldrick, 2008 ▶); software used to prepare material for publication: *SHELXTL*.

## Supplementary Material

Crystal structure: contains datablock(s) I, New_Global_Publ_Block. DOI: 10.1107/S1600536813002341/hg5286sup1.cif

Structure factors: contains datablock(s) I. DOI: 10.1107/S1600536813002341/hg5286Isup2.hkl

Additional supplementary materials:  crystallographic information; 3D view; checkCIF report

## Figures and Tables

**Table 1:** Hydrogen-bond geometry (Å, °)

*D*—H⋯*A*	*D*—H	H⋯*A*	*D*⋯*A*	*D*—H⋯*A*
N1*A*—H1*AA*⋯O2*A* ^i^	0.88	1.98	2.820 (3)	159
N1*B*—H1*BA*⋯O2*C*	0.88	2.01	2.849 (3)	159
N1*C*—H1*CA*⋯O2*B*	0.88	1.96	2.795 (3)	158
C11*A*—H11*C*⋯O2*B* ^ii^	0.98	2.39	3.344 (4)	163
C11*C*—H11*H*⋯O2*A* ^iii^	0.98	2.45	3.377 (4)	158
C12*C*—H12*G*⋯O2*A* ^iii^	0.98	2.44	3.186 (4)	133
C12*C*—H12*H*⋯O1*B* ^iii^	0.98	2.51	3.178 (4)	125
C12*A*—H12*A*⋯O2*B* ^ii^	0.98	2.50	3.273 (4)	136
C11*B*—H11*F*⋯O2*C* ^iv^	0.98	2.42	3.364 (4)	163
C12*B*—H12*D*⋯O2*C* ^iv^	0.98	2.46	3.282 (4)	142
C17*B*—H17*B*⋯Cl1*B* ^v^	0.95	2.89	3.705 (4)	144

Symmetry codes: (i) 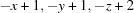; (ii) 
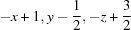
; (iii) 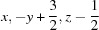; (iv) 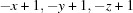; (v) 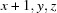.

**Fig. 1.**
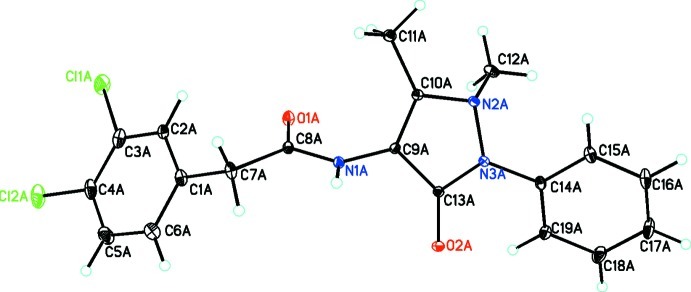
The molecular structure of the title molecule with the atom numbering. The displacement ellipsoids are drawn at the 30% probability level.

**Fig. 2.**
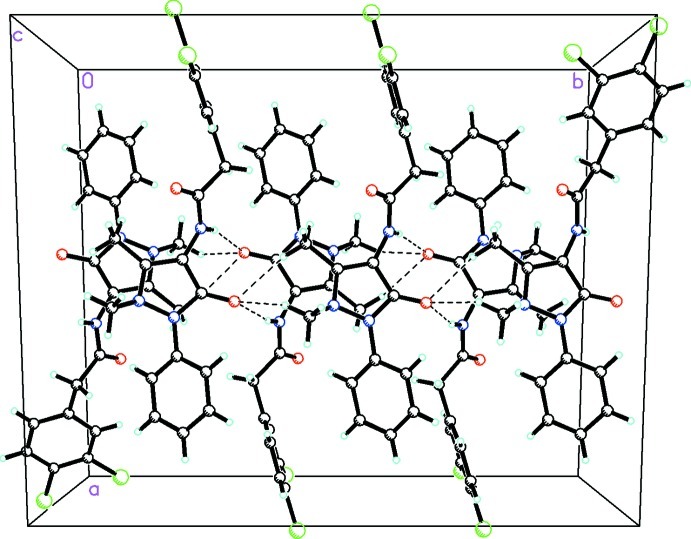
The crystal packing of the title compound, viewed along the b axis. Hydrogen bonds are shown as dashed lines - see Table 1 for details.

## supplementary crystallographic information

### Comment

N-Substituted 2-arylacetamides are very interesting compounds because of their structural similarity to the lateral chain of natural benzylpenicillin (Mijin *et al.*, 2006, 2008). Amides are also used as ligands due to their excellent coordination abilities (Wu *et al.*, 2008, 2010). Crystal structures of some acetamide derivatives *viz*., (2E)-1-(2,5-dimethoxyphenyl)-3-(3-nitrophenyl)prop-2-en-1-one, *N*-(4-bromophenyl)-2-(naphthalen-1-yl)acetamide, *N*-(1,5-dimethyl-3-oxo-2-phenyl-2,3-dihydro-1*H*-pyrazol-4-yl)-2-[4-(methylsulfanyl)phenyl]acetamide, *N*-(4-bromophenyl)-2-(4-chlorophenyl)acetamide (Fun *et al.*, 2011*a*, 2011*b*, 2012*a*, 2012*b*) have been reported. Two related molecules with different dichloro substitution patterns have recently been published (Butcher *et al.*, 2013*a*, 2013*b*). In view of the importance of amides we report herein the crystal structure of the title compound (I).

In the title compound, C_19_H_17_Cl_2_N_3_O_2_, there are three molecules in the asymmetric unit and each differs in the comformation adopted. In each molecule the amide group is planar but two of the molecules (B and C) are linked through N—H···O hydrogen bonding to an adjoining molecule forming dimers of the *R*_2_^2^(10) type (Bernstein *et al.*, 1995) while molecule A forms such dimers with itself. The major conformational difference between three molecules is seen in the dihedral angles between the dichlorophenyl and 2,3-dihydro-1*H*-pyrazol-4-yl rings which are 54.8 (2)°, 76.2 (2),° and 77.5 (2)° for A, B, and C, respectively. Due to steric repulsion the amide group is rotated with respect to both the dichlorophenyl and 2,3-dihydro-1*H*-pyrazol-4-yl rings with dihedral angles of 44.5 (2)° and 56.2 (2)° for A; 51.1 (2) and 54.1 (2) for B; and 53.8 (2) and 54.6 (2) for C. All other metrical parameters are in the normal ranges (Allen, 2002).

### Experimental

3,4-Dichlorophenylacetic acid (0.240 g, 1 mmol) and 4-aminoantipyrine (0.203 g, 1 mmol), 1-ethyl-3-(3-dimethylaminopropyl)-carbodiimide hydrochloride (1.0 g, 0.01 mol) and were dissolved in dichloromethane (20 ml). The mixture was stirred in presence of triethylamine at 273 K for about 3 h. The contents were poured into 100 ml of ice-cold aqueous hydrochloric acid with stirring, which was extracted thrice with dichloromethane. The organic layer was washed with saturated NaHCO_3_ solution and brine solution, dried and concentrated under reduced pressure to give the title compound (I). Single crystals were grown from methylene chloride by the slow evaporation method (m.p.: 473–475 K).

### Refinement

The H atoms were placed in calculated positions and refined in the riding mode: N—H = 0.88 Å, C—H = 0.95–0.99 Å with *U*_iso_(H) = 1.5*U*_eq_(C) for methyl H atoms and = 1.2*U*_eq_(O,*C*) for other H atoms.

### Crystal data

**Table:**

C_19_H_17_Cl_2_N_3_O_2_	*F*(000) = 2424
*M**_r_* = 390.26	*D*_x_ = 1.419 Mg m^−^^3^
Monoclinic, *P*2_1_/*c*	Mo *K*α radiation, λ = 0.71073 Å
Hall symbol: -P 2ybc	Cell parameters from 4632 reflections
*a* = 17.2064 (8) Å	θ = 3.1–37.6°
*b* = 20.7984 (9) Å	µ = 0.37 mm^−^^1^
*c* = 15.6102 (7) Å	*T* = 123 K
β = 101.213 (4)°	Prism, colorless
*V* = 5479.7 (4) Å^3^	0.51 × 0.34 × 0.10 mm
*Z* = 12	

### Data collection

**Table:**

Agilent Xcalibur (Ruby, Gemini) diffractometer	27521 independent reflections
Radiation source: Enhance (Mo) X-ray Source	11938 reflections with *I* > 2σ(*I*)
Graphite monochromator	*R*_int_ = 0.076
Detector resolution: 10.5081 pixels mm^-1^	θ_max_ = 37.7°, θ_min_ = 3.1°
ω scans	*h* = −28→25
Absorption correction: analytical [*CrysAlis PRO* (Agilent, 2011) based on expressions derived by Clark & Reid (1995)]	*k* = −35→25
*T*_min_ = 0.743, *T*_max_ = 0.932	*l* = −26→23
54403 measured reflections	

### Refinement

**Table:**

Refinement on *F*^2^	Primary atom site location: structure-invariant direct methods
Least-squares matrix: full	Secondary atom site location: difference Fourier map
*R*[*F*^2^ > 2σ(*F*^2^)] = 0.119	Hydrogen site location: inferred from neighbouring sites
*wR*(*F*^2^) = 0.345	H-atom parameters constrained
*S* = 1.02	*w* = 1/[σ^2^(*F*_o_^2^) + (0.1574*P*)^2^] where *P* = (*F*_o_^2^ + 2*F*_c_^2^)/3
27521 reflections	(Δ/σ)_max_ = 0.001
709 parameters	Δρ_max_ = 3.46 e Å^−^^3^
0 restraints	Δρ_min_ = −0.84 e Å^−^^3^

### Special details

**Table:**

Experimental. Analytical numeric absorption correction using a multifaceted crystal model based on expressions derived by R.C. Clark & J.S. Reid. (Clark, R. C. & Reid, J. S. (1995). Acta Cryst. A51, 887-897)
Geometry. All e.s.d.'s (except the e.s.d. in the dihedral angle between two l.s. planes) are estimated using the full covariance matrix. The cell e.s.d.'s are taken into account individually in the estimation of e.s.d.'s in distances, angles and torsion angles; correlations between e.s.d.'s in cell parameters are only used when they are defined by crystal symmetry. An approximate (isotropic) treatment of cell e.s.d.'s is used for estimating e.s.d.'s involving l.s. planes.
Refinement. Refinement of *F*^2^ against ALL reflections. The weighted *R*-factor *wR* and goodness of fit *S* are based on *F*^2^, conventional *R*-factors *R* are based on *F*, with *F* set to zero for negative *F*^2^. The threshold expression of *F*^2^ > σ(*F*^2^) is used only for calculating *R*-factors(gt) *etc*. and is not relevant to the choice of reflections for refinement. *R*-factors based on *F*^2^ are statistically about twice as large as those based on *F*, and *R*- factors based on ALL data will be even larger.

### Fractional atomic coordinates and isotropic or equivalent isotropic displacement parameters (Å^2^)

**Table:**

	*x*	*y*	*z*	*U*_iso_*/*U*_eq_	
Cl1A	0.96725 (6)	0.40403 (6)	0.84456 (8)	0.0462 (3)	
Cl2A	1.02211 (6)	0.54928 (7)	0.85730 (7)	0.0502 (3)	
O1A	0.68657 (13)	0.39601 (11)	0.88454 (14)	0.0210 (4)	
O2A	0.44835 (12)	0.49727 (10)	0.89719 (13)	0.0174 (4)	
N1A	0.60317 (14)	0.43172 (11)	0.97043 (16)	0.0157 (4)	
H1AA	0.5996	0.4529	1.0183	0.019*	
N2A	0.44192 (14)	0.33263 (11)	0.85826 (16)	0.0166 (4)	
N3A	0.40983 (14)	0.39502 (11)	0.84534 (16)	0.0160 (4)	
C1A	0.80792 (19)	0.48640 (17)	0.9654 (2)	0.0260 (7)	
C2A	0.85147 (18)	0.44182 (16)	0.9279 (2)	0.0231 (6)	
H2AA	0.8365	0.3978	0.9258	0.028*	
C3A	0.9165 (2)	0.4607 (2)	0.8933 (2)	0.0343 (8)	
C4A	0.9403 (2)	0.5249 (2)	0.8975 (2)	0.0342 (8)	
C5A	0.8975 (2)	0.5697 (2)	0.9352 (3)	0.0380 (9)	
H5AA	0.9137	0.6134	0.9391	0.046*	
C6A	0.8306 (2)	0.55102 (19)	0.9675 (2)	0.0334 (8)	
H6AA	0.8005	0.5824	0.9910	0.040*	
C7A	0.73992 (18)	0.46564 (17)	1.0061 (2)	0.0234 (6)	
H7AA	0.7160	0.5044	1.0271	0.028*	
H7AB	0.7610	0.4388	1.0578	0.028*	
C8A	0.67481 (16)	0.42806 (14)	0.94674 (17)	0.0152 (5)	
C9A	0.53436 (15)	0.40317 (13)	0.92196 (17)	0.0143 (5)	
C10A	0.51996 (16)	0.34018 (13)	0.90096 (17)	0.0142 (5)	
C11A	0.57441 (18)	0.28372 (14)	0.9175 (2)	0.0201 (5)	
H11A	0.6141	0.2911	0.9708	0.030*	
H11B	0.6011	0.2780	0.8680	0.030*	
H11C	0.5438	0.2450	0.9246	0.030*	
C12A	0.4225 (2)	0.29090 (15)	0.7810 (2)	0.0230 (6)	
H12A	0.4475	0.2488	0.7943	0.034*	
H12B	0.4422	0.3105	0.7322	0.034*	
H12C	0.3649	0.2856	0.7649	0.034*	
C13A	0.46347 (16)	0.43918 (13)	0.88913 (17)	0.0136 (5)	
C14A	0.32692 (17)	0.40479 (15)	0.82140 (18)	0.0172 (5)	
C15A	0.27399 (19)	0.36295 (17)	0.8493 (2)	0.0234 (6)	
H15A	0.2926	0.3266	0.8841	0.028*	
C16A	0.1929 (2)	0.3754 (2)	0.8251 (2)	0.0332 (8)	
H16A	0.1561	0.3467	0.8431	0.040*	
C17A	0.1657 (2)	0.4279 (2)	0.7762 (3)	0.0367 (9)	
H17A	0.1104	0.4361	0.7615	0.044*	
C18A	0.2186 (2)	0.4698 (2)	0.7476 (2)	0.0334 (8)	
H18A	0.1993	0.5059	0.7127	0.040*	
C19A	0.29997 (18)	0.45836 (16)	0.7703 (2)	0.0223 (6)	
H19A	0.3365	0.4867	0.7513	0.027*	
Cl1B	0.03779 (6)	0.59135 (6)	0.65631 (7)	0.0425 (3)	
Cl2B	−0.07149 (5)	0.56646 (5)	0.47093 (8)	0.0413 (3)	
O1B	0.31484 (12)	0.56614 (11)	0.61156 (14)	0.0214 (4)	
O2B	0.55541 (12)	0.66284 (9)	0.59479 (13)	0.0168 (4)	
N1B	0.40028 (14)	0.59847 (11)	0.52644 (15)	0.0149 (4)	
H1BA	0.4040	0.6174	0.4770	0.018*	
N2B	0.56017 (14)	0.50006 (11)	0.64305 (15)	0.0158 (4)	
N3B	0.59287 (14)	0.56181 (11)	0.65139 (16)	0.0154 (4)	
C1B	0.18424 (19)	0.62618 (16)	0.4936 (2)	0.0252 (6)	
C2B	0.15246 (18)	0.61974 (16)	0.5682 (2)	0.0235 (6)	
H2BA	0.1846	0.6277	0.6239	0.028*	
C3B	0.0743 (2)	0.60175 (17)	0.5619 (2)	0.0291 (7)	
C4B	0.02603 (19)	0.59001 (16)	0.4803 (2)	0.0282 (7)	
C5B	0.0574 (2)	0.59658 (19)	0.4055 (3)	0.0339 (8)	
H5BA	0.0249	0.5893	0.3498	0.041*	
C6B	0.1362 (2)	0.61376 (18)	0.4118 (2)	0.0296 (7)	
H6BA	0.1578	0.6171	0.3604	0.035*	
C7B	0.26904 (18)	0.64545 (17)	0.4993 (2)	0.0266 (7)	
H7BA	0.2772	0.6885	0.5268	0.032*	
H7BB	0.2797	0.6491	0.4394	0.032*	
C8B	0.32919 (16)	0.59851 (14)	0.55152 (18)	0.0159 (5)	
C9B	0.46845 (16)	0.56950 (13)	0.57595 (17)	0.0136 (5)	
C10B	0.48187 (16)	0.50685 (13)	0.60107 (18)	0.0147 (5)	
C11B	0.42690 (18)	0.45144 (14)	0.5903 (2)	0.0196 (5)	
H11D	0.3853	0.4580	0.5385	0.029*	
H11E	0.4028	0.4476	0.6420	0.029*	
H11F	0.4562	0.4120	0.5832	0.029*	
C12B	0.5825 (2)	0.45914 (14)	0.72096 (19)	0.0218 (6)	
H12D	0.5588	0.4165	0.7090	0.033*	
H12E	0.5633	0.4787	0.7701	0.033*	
H12F	0.6403	0.4551	0.7357	0.033*	
C13B	0.53967 (16)	0.60554 (13)	0.60611 (17)	0.0133 (5)	
C14B	0.67664 (17)	0.57215 (14)	0.67190 (18)	0.0167 (5)	
C15B	0.72831 (19)	0.53193 (17)	0.6391 (2)	0.0251 (6)	
H15B	0.7090	0.4955	0.6048	0.030*	
C16B	0.8090 (2)	0.5461 (2)	0.6575 (3)	0.0346 (8)	
H16B	0.8452	0.5191	0.6356	0.042*	
C17B	0.8366 (2)	0.5992 (2)	0.7076 (3)	0.0343 (8)	
H17B	0.8916	0.6092	0.7187	0.041*	
C18B	0.7847 (2)	0.63768 (19)	0.7415 (2)	0.0300 (7)	
H18B	0.8043	0.6734	0.7770	0.036*	
C19B	0.70414 (17)	0.62481 (16)	0.72430 (19)	0.0209 (6)	
H19B	0.6684	0.6513	0.7477	0.025*	
Cl1C	0.95668 (6)	0.74477 (6)	0.30440 (7)	0.0418 (3)	
Cl2C	1.07466 (6)	0.77783 (5)	0.48017 (8)	0.0445 (3)	
O1C	0.68889 (12)	0.77189 (11)	0.37835 (14)	0.0199 (4)	
O2C	0.44782 (12)	0.67409 (10)	0.39335 (13)	0.0177 (4)	
N1C	0.60697 (14)	0.73495 (12)	0.46543 (16)	0.0165 (4)	
H1CA	0.6041	0.7136	0.5134	0.020*	
N2C	0.44993 (14)	0.83872 (11)	0.35446 (15)	0.0148 (4)	
N3C	0.41482 (14)	0.77732 (12)	0.34240 (16)	0.0153 (4)	
C1C	0.82344 (19)	0.71341 (16)	0.4839 (2)	0.0249 (6)	
C2C	0.85001 (18)	0.71832 (16)	0.4053 (2)	0.0249 (6)	
H2CA	0.8151	0.7079	0.3521	0.030*	
C3C	0.92665 (19)	0.73824 (17)	0.4035 (2)	0.0279 (7)	
C4C	0.9792 (2)	0.75366 (17)	0.4810 (3)	0.0304 (7)	
C5C	0.9537 (2)	0.74789 (18)	0.5595 (3)	0.0317 (8)	
H5CA	0.9890	0.7571	0.6129	0.038*	
C6C	0.8762 (2)	0.72867 (18)	0.5603 (2)	0.0288 (7)	
H6CA	0.8591	0.7259	0.6145	0.035*	
C7C	0.74038 (17)	0.69158 (16)	0.4860 (2)	0.0232 (6)	
H7CA	0.7343	0.6877	0.5475	0.028*	
H7CB	0.7319	0.6485	0.4589	0.028*	
C8C	0.67712 (16)	0.73747 (13)	0.43810 (18)	0.0158 (5)	
C9C	0.53850 (16)	0.76571 (13)	0.41904 (17)	0.0134 (5)	
C10C	0.52724 (16)	0.82917 (13)	0.39794 (17)	0.0143 (5)	
C11C	0.58400 (17)	0.88362 (14)	0.4159 (2)	0.0187 (5)	
H11G	0.6231	0.8747	0.4692	0.028*	
H11H	0.5552	0.9232	0.4236	0.028*	
H11I	0.6112	0.8888	0.3667	0.028*	
C12C	0.4357 (2)	0.87879 (15)	0.27529 (19)	0.0226 (6)	
H12G	0.4607	0.9209	0.2886	0.034*	
H12H	0.3785	0.8844	0.2549	0.034*	
H12I	0.4584	0.8578	0.2296	0.034*	
C13C	0.46585 (16)	0.73144 (14)	0.38594 (17)	0.0146 (5)	
C14C	0.33155 (17)	0.77181 (15)	0.31674 (19)	0.0183 (5)	
C15C	0.28252 (19)	0.81775 (18)	0.3418 (2)	0.0266 (7)	
H15C	0.3044	0.8532	0.3768	0.032*	
C16C	0.2006 (2)	0.8117 (2)	0.3154 (3)	0.0416 (10)	
H16C	0.1665	0.8434	0.3318	0.050*	
C17C	0.1691 (2)	0.7599 (2)	0.2656 (3)	0.0464 (11)	
H17C	0.1133	0.7559	0.2476	0.056*	
C18C	0.2190 (2)	0.7133 (2)	0.2414 (2)	0.0358 (9)	
H18C	0.1968	0.6775	0.2076	0.043*	
C19C	0.30039 (18)	0.71880 (16)	0.2661 (2)	0.0222 (6)	
H19C	0.3344	0.6873	0.2492	0.027*	

### Atomic displacement parameters (Å^2^)

**Table:**

	*U*^11^	*U*^22^	*U*^33^	*U*^12^	*U*^13^	*U*^23^
Cl1A	0.0284 (5)	0.0573 (7)	0.0586 (7)	−0.0007 (4)	0.0225 (5)	−0.0106 (5)
Cl2A	0.0303 (5)	0.0761 (8)	0.0440 (6)	−0.0183 (5)	0.0069 (4)	0.0174 (5)
O1A	0.0171 (9)	0.0288 (12)	0.0183 (9)	−0.0018 (8)	0.0063 (8)	−0.0073 (9)
O2A	0.0188 (9)	0.0130 (9)	0.0207 (9)	0.0011 (7)	0.0048 (8)	−0.0006 (8)
N1A	0.0146 (10)	0.0155 (11)	0.0174 (10)	−0.0014 (8)	0.0038 (8)	−0.0040 (9)
N2A	0.0176 (10)	0.0135 (11)	0.0192 (11)	−0.0011 (8)	0.0052 (9)	0.0002 (9)
N3A	0.0152 (10)	0.0132 (11)	0.0194 (11)	−0.0019 (8)	0.0027 (9)	−0.0005 (9)
C1A	0.0208 (14)	0.0319 (18)	0.0239 (15)	−0.0060 (12)	0.0010 (12)	−0.0019 (13)
C2A	0.0154 (12)	0.0251 (15)	0.0290 (15)	−0.0030 (11)	0.0047 (11)	0.0008 (13)
C3A	0.0208 (15)	0.052 (2)	0.0297 (17)	−0.0008 (15)	0.0039 (13)	0.0015 (17)
C4A	0.0221 (15)	0.051 (2)	0.0276 (16)	−0.0131 (15)	0.0010 (13)	0.0110 (16)
C5A	0.038 (2)	0.032 (2)	0.043 (2)	−0.0128 (16)	0.0051 (17)	0.0101 (17)
C6A	0.0343 (19)	0.0319 (19)	0.0338 (18)	−0.0052 (15)	0.0065 (15)	−0.0017 (15)
C7A	0.0182 (13)	0.0321 (17)	0.0197 (13)	−0.0086 (12)	0.0035 (11)	−0.0027 (12)
C8A	0.0137 (11)	0.0178 (13)	0.0143 (11)	−0.0019 (9)	0.0035 (9)	0.0007 (10)
C9A	0.0125 (10)	0.0147 (12)	0.0158 (11)	0.0010 (9)	0.0030 (9)	0.0013 (10)
C10A	0.0163 (11)	0.0145 (12)	0.0124 (11)	0.0007 (9)	0.0046 (9)	−0.0008 (9)
C11A	0.0212 (13)	0.0168 (13)	0.0239 (14)	0.0001 (10)	0.0082 (11)	0.0010 (11)
C12A	0.0327 (16)	0.0170 (14)	0.0183 (13)	−0.0042 (12)	0.0028 (12)	−0.0053 (11)
C13A	0.0144 (11)	0.0131 (12)	0.0137 (11)	−0.0005 (9)	0.0038 (9)	−0.0001 (9)
C14A	0.0155 (11)	0.0218 (14)	0.0140 (11)	−0.0041 (10)	0.0019 (9)	−0.0018 (10)
C15A	0.0210 (14)	0.0300 (16)	0.0190 (13)	−0.0073 (12)	0.0031 (11)	0.0016 (12)
C16A	0.0180 (14)	0.051 (2)	0.0321 (17)	−0.0102 (14)	0.0073 (13)	0.0029 (17)
C17A	0.0133 (13)	0.059 (3)	0.0374 (19)	−0.0007 (15)	0.0029 (13)	0.0074 (18)
C18A	0.0220 (15)	0.041 (2)	0.0350 (18)	0.0077 (14)	0.0009 (14)	0.0092 (16)
C19A	0.0180 (13)	0.0274 (16)	0.0212 (13)	0.0014 (11)	0.0035 (11)	0.0024 (12)
Cl1B	0.0258 (4)	0.0647 (7)	0.0410 (5)	0.0036 (4)	0.0167 (4)	0.0069 (5)
Cl2B	0.0203 (4)	0.0342 (5)	0.0670 (7)	0.0011 (3)	0.0027 (4)	0.0015 (5)
O1B	0.0162 (9)	0.0290 (12)	0.0198 (10)	−0.0004 (8)	0.0057 (8)	0.0062 (9)
O2B	0.0181 (9)	0.0124 (9)	0.0208 (9)	−0.0001 (7)	0.0056 (8)	0.0011 (8)
N1B	0.0141 (10)	0.0167 (11)	0.0146 (10)	0.0005 (8)	0.0046 (8)	0.0048 (9)
N2B	0.0175 (10)	0.0117 (10)	0.0178 (10)	−0.0001 (8)	0.0025 (9)	0.0008 (9)
N3B	0.0126 (9)	0.0137 (10)	0.0189 (10)	0.0009 (8)	0.0007 (8)	0.0004 (9)
C1B	0.0196 (14)	0.0250 (16)	0.0308 (16)	0.0074 (12)	0.0042 (12)	0.0083 (13)
C2B	0.0170 (13)	0.0286 (16)	0.0253 (14)	0.0057 (11)	0.0048 (11)	0.0009 (13)
C3B	0.0211 (15)	0.0292 (17)	0.0376 (18)	0.0044 (13)	0.0069 (13)	0.0045 (15)
C4B	0.0174 (13)	0.0233 (16)	0.0427 (19)	0.0032 (11)	0.0028 (13)	0.0007 (15)
C5B	0.0255 (16)	0.036 (2)	0.0376 (19)	0.0051 (14)	−0.0010 (15)	−0.0018 (16)
C6B	0.0270 (16)	0.0317 (18)	0.0294 (16)	0.0054 (13)	0.0040 (14)	0.0027 (14)
C7B	0.0188 (14)	0.0279 (16)	0.0343 (17)	0.0064 (12)	0.0080 (12)	0.0146 (14)
C8B	0.0137 (11)	0.0172 (13)	0.0167 (12)	−0.0007 (9)	0.0027 (9)	0.0027 (10)
C9B	0.0136 (11)	0.0141 (12)	0.0137 (11)	−0.0002 (9)	0.0040 (9)	0.0021 (9)
C10B	0.0149 (11)	0.0140 (12)	0.0163 (11)	0.0003 (9)	0.0058 (9)	−0.0003 (10)
C11B	0.0208 (13)	0.0140 (12)	0.0249 (14)	−0.0014 (10)	0.0070 (11)	−0.0018 (11)
C12B	0.0311 (16)	0.0143 (13)	0.0182 (13)	0.0005 (11)	0.0000 (12)	0.0052 (11)
C13B	0.0155 (11)	0.0128 (12)	0.0127 (11)	0.0013 (9)	0.0050 (9)	0.0001 (9)
C14B	0.0149 (11)	0.0199 (13)	0.0146 (11)	0.0020 (10)	0.0011 (9)	0.0009 (10)
C15B	0.0192 (13)	0.0280 (16)	0.0283 (15)	0.0072 (12)	0.0049 (12)	−0.0054 (13)
C16B	0.0200 (15)	0.042 (2)	0.043 (2)	0.0097 (14)	0.0095 (14)	−0.0020 (17)
C17B	0.0152 (14)	0.044 (2)	0.043 (2)	0.0000 (14)	0.0029 (14)	0.0046 (17)
C18B	0.0219 (15)	0.0368 (19)	0.0291 (16)	−0.0074 (13)	−0.0005 (13)	−0.0029 (15)
C19B	0.0164 (12)	0.0272 (15)	0.0186 (13)	0.0007 (11)	0.0024 (10)	−0.0022 (12)
Cl1C	0.0249 (4)	0.0613 (7)	0.0422 (5)	0.0078 (4)	0.0135 (4)	0.0122 (5)
Cl2C	0.0220 (4)	0.0449 (6)	0.0663 (7)	−0.0046 (4)	0.0080 (4)	−0.0016 (5)
O1C	0.0166 (9)	0.0238 (11)	0.0206 (10)	0.0024 (8)	0.0065 (8)	0.0072 (8)
O2C	0.0207 (10)	0.0123 (9)	0.0207 (10)	−0.0022 (7)	0.0054 (8)	0.0009 (8)
N1C	0.0165 (10)	0.0167 (11)	0.0169 (10)	0.0024 (8)	0.0047 (9)	0.0058 (9)
N2C	0.0147 (10)	0.0141 (11)	0.0153 (10)	0.0016 (8)	0.0018 (8)	0.0008 (8)
N3C	0.0127 (9)	0.0161 (11)	0.0172 (10)	−0.0001 (8)	0.0029 (8)	0.0017 (9)
C1C	0.0189 (13)	0.0254 (16)	0.0295 (16)	0.0065 (11)	0.0020 (12)	0.0055 (13)
C2C	0.0153 (13)	0.0280 (16)	0.0312 (16)	0.0034 (11)	0.0037 (12)	0.0039 (14)
C3C	0.0160 (13)	0.0318 (18)	0.0360 (18)	0.0071 (12)	0.0053 (12)	0.0059 (15)
C4C	0.0190 (14)	0.0248 (16)	0.048 (2)	0.0027 (12)	0.0067 (14)	0.0030 (15)
C5C	0.0240 (16)	0.0319 (18)	0.0371 (19)	0.0006 (13)	0.0005 (14)	−0.0017 (15)
C6C	0.0254 (16)	0.0325 (18)	0.0270 (16)	0.0048 (13)	0.0011 (13)	0.0000 (14)
C7C	0.0150 (12)	0.0229 (15)	0.0324 (16)	0.0053 (10)	0.0066 (11)	0.0108 (13)
C8C	0.0141 (11)	0.0158 (12)	0.0176 (12)	0.0012 (9)	0.0034 (9)	0.0005 (10)
C9C	0.0170 (11)	0.0125 (11)	0.0123 (10)	0.0017 (9)	0.0065 (9)	0.0011 (9)
C10C	0.0139 (11)	0.0148 (12)	0.0150 (11)	0.0003 (9)	0.0048 (9)	−0.0012 (10)
C11C	0.0201 (13)	0.0132 (12)	0.0236 (13)	−0.0015 (10)	0.0061 (11)	−0.0012 (11)
C12C	0.0326 (16)	0.0183 (14)	0.0153 (12)	0.0018 (12)	0.0006 (12)	0.0043 (11)
C13C	0.0142 (11)	0.0166 (12)	0.0139 (11)	0.0021 (9)	0.0052 (9)	0.0009 (10)
C14C	0.0160 (12)	0.0228 (14)	0.0162 (12)	0.0009 (10)	0.0033 (10)	0.0022 (11)
C15C	0.0188 (13)	0.0335 (18)	0.0267 (15)	0.0070 (12)	0.0025 (12)	−0.0023 (14)
C16C	0.0230 (17)	0.060 (3)	0.043 (2)	0.0134 (17)	0.0081 (16)	−0.008 (2)
C17C	0.0173 (16)	0.073 (3)	0.046 (2)	−0.0040 (18)	−0.0006 (16)	0.000 (2)
C18C	0.0232 (16)	0.052 (2)	0.0305 (17)	−0.0131 (16)	0.0020 (14)	−0.0031 (17)
C19C	0.0201 (13)	0.0289 (16)	0.0179 (13)	−0.0048 (12)	0.0042 (11)	−0.0021 (12)

### Geometric parameters (Å, º)

**Table:**

Cl1A—C3A	1.729 (4)	C7B—H7BA	0.9900
Cl2A—C4A	1.726 (4)	C7B—H7BB	0.9900
O1A—C8A	1.227 (3)	C9B—C10B	1.367 (4)
O2A—C13A	1.247 (3)	C9B—C13B	1.435 (4)
N1A—C8A	1.356 (3)	C10B—C11B	1.480 (4)
N1A—C9A	1.406 (4)	C11B—H11D	0.9800
N1A—H1AA	0.8800	C11B—H11E	0.9800
N2A—C10A	1.387 (4)	C11B—H11F	0.9800
N2A—N3A	1.409 (3)	C12B—H12D	0.9800
N2A—C12A	1.471 (4)	C12B—H12E	0.9800
N3A—C13A	1.385 (4)	C12B—H12F	0.9800
N3A—C14A	1.417 (4)	C14B—C15B	1.389 (4)
C1A—C2A	1.392 (5)	C14B—C19B	1.394 (4)
C1A—C6A	1.398 (5)	C15B—C16B	1.394 (5)
C1A—C7A	1.499 (4)	C15B—H15B	0.9500
C2A—C3A	1.389 (5)	C16B—C17B	1.383 (6)
C2A—H2AA	0.9500	C16B—H16B	0.9500
C3A—C4A	1.395 (6)	C17B—C18B	1.380 (5)
C4A—C5A	1.387 (6)	C17B—H17B	0.9500
C5A—C6A	1.398 (5)	C18B—C19B	1.385 (4)
C5A—H5AA	0.9500	C18B—H18B	0.9500
C6A—H6AA	0.9500	C19B—H19B	0.9500
C7A—C8A	1.523 (4)	Cl1C—C3C	1.728 (4)
C7A—H7AA	0.9900	Cl2C—C4C	1.720 (4)
C7A—H7AB	0.9900	O1C—C8C	1.224 (3)
C9A—C10A	1.361 (4)	O2C—C13C	1.244 (3)
C9A—C13A	1.438 (4)	N1C—C8C	1.357 (4)
C10A—C11A	1.493 (4)	N1C—C9C	1.411 (4)
C11A—H11A	0.9800	N1C—H1CA	0.8800
C11A—H11B	0.9800	N2C—C10C	1.385 (4)
C11A—H11C	0.9800	N2C—N3C	1.410 (3)
C12A—H12A	0.9800	N2C—C12C	1.471 (4)
C12A—H12B	0.9800	N3C—C13C	1.383 (4)
C12A—H12C	0.9800	N3C—C14C	1.415 (4)
C14A—C15A	1.389 (4)	C1C—C6C	1.388 (5)
C14A—C19A	1.396 (4)	C1C—C2C	1.394 (5)
C15A—C16A	1.397 (5)	C1C—C7C	1.506 (4)
C15A—H15A	0.9500	C2C—C3C	1.388 (5)
C16A—C17A	1.363 (6)	C2C—H2CA	0.9500
C16A—H16A	0.9500	C3C—C4C	1.399 (5)
C17A—C18A	1.393 (5)	C4C—C5C	1.386 (6)
C17A—H17A	0.9500	C5C—C6C	1.394 (5)
C18A—C19A	1.396 (5)	C5C—H5CA	0.9500
C18A—H18A	0.9500	C6C—H6CA	0.9500
C19A—H19A	0.9500	C7C—C8C	1.529 (4)
Cl1B—C3B	1.725 (4)	C7C—H7CA	0.9900
Cl2B—C4B	1.727 (3)	C7C—H7CB	0.9900
O1B—C8B	1.218 (3)	C9C—C10C	1.365 (4)
O2B—C13B	1.242 (3)	C9C—C13C	1.444 (4)
N1B—C8B	1.355 (3)	C10C—C11C	1.486 (4)
N1B—C9B	1.408 (3)	C11C—H11G	0.9800
N1B—H1BA	0.8800	C11C—H11H	0.9800
N2B—C10B	1.386 (4)	C11C—H11I	0.9800
N2B—N3B	1.398 (3)	C12C—H12G	0.9800
N2B—C12B	1.473 (4)	C12C—H12H	0.9800
N3B—C13B	1.383 (3)	C12C—H12I	0.9800
N3B—C14B	1.431 (4)	C14C—C15C	1.381 (4)
C1B—C2B	1.386 (5)	C14C—C19C	1.401 (4)
C1B—C6B	1.403 (5)	C15C—C16C	1.396 (5)
C1B—C7B	1.499 (5)	C15C—H15C	0.9500
C2B—C3B	1.381 (5)	C16C—C17C	1.376 (6)
C2B—H2BA	0.9500	C16C—H16C	0.9500
C3B—C4B	1.401 (5)	C17C—C18C	1.394 (6)
C4B—C5B	1.384 (5)	C17C—H17C	0.9500
C5B—C6B	1.388 (5)	C18C—C19C	1.384 (5)
C5B—H5BA	0.9500	C18C—H18C	0.9500
C6B—H6BA	0.9500	C19C—H19C	0.9500
C7B—C8B	1.536 (4)		
			
C8A—N1A—C9A	123.0 (2)	N1B—C9B—C13B	121.6 (2)
C8A—N1A—H1AA	118.5	C9B—C10B—N2B	109.1 (2)
C9A—N1A—H1AA	118.5	C9B—C10B—C11B	129.9 (3)
C10A—N2A—N3A	106.3 (2)	N2B—C10B—C11B	121.0 (2)
C10A—N2A—C12A	120.3 (2)	C10B—C11B—H11D	109.5
N3A—N2A—C12A	113.9 (2)	C10B—C11B—H11E	109.5
C13A—N3A—N2A	109.6 (2)	H11D—C11B—H11E	109.5
C13A—N3A—C14A	125.1 (2)	C10B—C11B—H11F	109.5
N2A—N3A—C14A	121.1 (2)	H11D—C11B—H11F	109.5
C2A—C1A—C6A	118.6 (3)	H11E—C11B—H11F	109.5
C2A—C1A—C7A	121.0 (3)	N2B—C12B—H12D	109.5
C6A—C1A—C7A	120.3 (3)	N2B—C12B—H12E	109.5
C3A—C2A—C1A	121.0 (3)	H12D—C12B—H12E	109.5
C3A—C2A—H2AA	119.5	N2B—C12B—H12F	109.5
C1A—C2A—H2AA	119.5	H12D—C12B—H12F	109.5
C2A—C3A—C4A	120.2 (4)	H12E—C12B—H12F	109.5
C2A—C3A—Cl1A	119.5 (3)	O2B—C13B—N3B	124.2 (3)
C4A—C3A—Cl1A	120.3 (3)	O2B—C13B—C9B	130.5 (3)
C5A—C4A—C3A	119.2 (3)	N3B—C13B—C9B	105.2 (2)
C5A—C4A—Cl2A	119.7 (3)	C15B—C14B—C19B	121.4 (3)
C3A—C4A—Cl2A	121.1 (3)	C15B—C14B—N3B	121.1 (3)
C4A—C5A—C6A	120.6 (4)	C19B—C14B—N3B	117.5 (3)
C4A—C5A—H5AA	119.7	C14B—C15B—C16B	118.7 (3)
C6A—C5A—H5AA	119.7	C14B—C15B—H15B	120.6
C5A—C6A—C1A	120.3 (4)	C16B—C15B—H15B	120.6
C5A—C6A—H6AA	119.8	C17B—C16B—C15B	120.3 (3)
C1A—C6A—H6AA	119.8	C17B—C16B—H16B	119.9
C1A—C7A—C8A	115.6 (3)	C15B—C16B—H16B	119.9
C1A—C7A—H7AA	108.4	C18B—C17B—C16B	120.2 (3)
C8A—C7A—H7AA	108.4	C18B—C17B—H17B	119.9
C1A—C7A—H7AB	108.4	C16B—C17B—H17B	119.9
C8A—C7A—H7AB	108.4	C17B—C18B—C19B	120.8 (3)
H7AA—C7A—H7AB	107.4	C17B—C18B—H18B	119.6
O1A—C8A—N1A	123.0 (3)	C19B—C18B—H18B	119.6
O1A—C8A—C7A	123.1 (3)	C18B—C19B—C14B	118.6 (3)
N1A—C8A—C7A	113.9 (2)	C18B—C19B—H19B	120.7
C10A—C9A—N1A	129.1 (3)	C14B—C19B—H19B	120.7
C10A—C9A—C13A	108.3 (2)	C8C—N1C—C9C	121.7 (2)
N1A—C9A—C13A	122.6 (2)	C8C—N1C—H1CA	119.1
C9A—C10A—N2A	109.8 (2)	C9C—N1C—H1CA	119.1
C9A—C10A—C11A	129.5 (3)	C10C—N2C—N3C	106.3 (2)
N2A—C10A—C11A	120.7 (2)	C10C—N2C—C12C	118.7 (2)
C10A—C11A—H11A	109.5	N3C—N2C—C12C	113.8 (2)
C10A—C11A—H11B	109.5	C13C—N3C—N2C	110.2 (2)
H11A—C11A—H11B	109.5	C13C—N3C—C14C	126.5 (2)
C10A—C11A—H11C	109.5	N2C—N3C—C14C	119.7 (2)
H11A—C11A—H11C	109.5	C6C—C1C—C2C	117.9 (3)
H11B—C11A—H11C	109.5	C6C—C1C—C7C	121.0 (3)
N2A—C12A—H12A	109.5	C2C—C1C—C7C	121.1 (3)
N2A—C12A—H12B	109.5	C3C—C2C—C1C	121.0 (3)
H12A—C12A—H12B	109.5	C3C—C2C—H2CA	119.5
N2A—C12A—H12C	109.5	C1C—C2C—H2CA	119.5
H12A—C12A—H12C	109.5	C2C—C3C—C4C	120.5 (3)
H12B—C12A—H12C	109.5	C2C—C3C—Cl1C	119.5 (3)
O2A—C13A—N3A	124.1 (3)	C4C—C3C—Cl1C	120.0 (3)
O2A—C13A—C9A	130.4 (3)	C5C—C4C—C3C	118.8 (3)
N3A—C13A—C9A	105.6 (2)	C5C—C4C—Cl2C	119.9 (3)
C15A—C14A—C19A	120.9 (3)	C3C—C4C—Cl2C	121.2 (3)
C15A—C14A—N3A	121.0 (3)	C4C—C5C—C6C	120.1 (3)
C19A—C14A—N3A	118.1 (3)	C4C—C5C—H5CA	120.0
C14A—C15A—C16A	118.7 (3)	C6C—C5C—H5CA	120.0
C14A—C15A—H15A	120.6	C1C—C6C—C5C	121.6 (3)
C16A—C15A—H15A	120.6	C1C—C6C—H6CA	119.2
C17A—C16A—C15A	121.0 (3)	C5C—C6C—H6CA	119.2
C17A—C16A—H16A	119.5	C1C—C7C—C8C	112.9 (3)
C15A—C16A—H16A	119.5	C1C—C7C—H7CA	109.0
C16A—C17A—C18A	120.4 (3)	C8C—C7C—H7CA	109.0
C16A—C17A—H17A	119.8	C1C—C7C—H7CB	109.0
C18A—C17A—H17A	119.8	C8C—C7C—H7CB	109.0
C17A—C18A—C19A	119.8 (3)	H7CA—C7C—H7CB	107.8
C17A—C18A—H18A	120.1	O1C—C8C—N1C	123.5 (3)
C19A—C18A—H18A	120.1	O1C—C8C—C7C	122.0 (3)
C14A—C19A—C18A	119.1 (3)	N1C—C8C—C7C	114.5 (2)
C14A—C19A—H19A	120.5	C10C—C9C—N1C	128.9 (3)
C18A—C19A—H19A	120.5	C10C—C9C—C13C	108.6 (2)
C8B—N1B—C9B	123.0 (2)	N1C—C9C—C13C	122.6 (2)
C8B—N1B—H1BA	118.5	C9C—C10C—N2C	109.6 (2)
C9B—N1B—H1BA	118.5	C9C—C10C—C11C	129.4 (3)
C10B—N2B—N3B	106.7 (2)	N2C—C10C—C11C	121.1 (2)
C10B—N2B—C12B	121.6 (2)	C10C—C11C—H11G	109.5
N3B—N2B—C12B	114.7 (2)	C10C—C11C—H11H	109.5
C13B—N3B—N2B	110.0 (2)	H11G—C11C—H11H	109.5
C13B—N3B—C14B	123.6 (2)	C10C—C11C—H11I	109.5
N2B—N3B—C14B	121.9 (2)	H11G—C11C—H11I	109.5
C2B—C1B—C6B	119.2 (3)	H11H—C11C—H11I	109.5
C2B—C1B—C7B	121.0 (3)	N2C—C12C—H12G	109.5
C6B—C1B—C7B	119.8 (3)	N2C—C12C—H12H	109.5
C3B—C2B—C1B	120.2 (3)	H12G—C12C—H12H	109.5
C3B—C2B—H2BA	119.9	N2C—C12C—H12I	109.5
C1B—C2B—H2BA	119.9	H12G—C12C—H12I	109.5
C2B—C3B—C4B	120.6 (3)	H12H—C12C—H12I	109.5
C2B—C3B—Cl1B	119.0 (3)	O2C—C13C—N3C	124.2 (3)
C4B—C3B—Cl1B	120.4 (3)	O2C—C13C—C9C	130.9 (3)
C5B—C4B—C3B	119.3 (3)	N3C—C13C—C9C	105.0 (2)
C5B—C4B—Cl2B	119.3 (3)	C15C—C14C—C19C	121.1 (3)
C3B—C4B—Cl2B	121.4 (3)	C15C—C14C—N3C	120.3 (3)
C4B—C5B—C6B	120.1 (3)	C19C—C14C—N3C	118.6 (3)
C4B—C5B—H5BA	119.9	C14C—C15C—C16C	119.5 (3)
C6B—C5B—H5BA	119.9	C14C—C15C—H15C	120.3
C5B—C6B—C1B	120.4 (3)	C16C—C15C—H15C	120.3
C5B—C6B—H6BA	119.8	C17C—C16C—C15C	120.1 (4)
C1B—C6B—H6BA	119.8	C17C—C16C—H16C	120.0
C1B—C7B—C8B	114.1 (3)	C15C—C16C—H16C	120.0
C1B—C7B—H7BA	108.7	C16C—C17C—C18C	120.2 (4)
C8B—C7B—H7BA	108.7	C16C—C17C—H17C	119.9
C1B—C7B—H7BB	108.7	C18C—C17C—H17C	119.9
C8B—C7B—H7BB	108.7	C19C—C18C—C17C	120.7 (4)
H7BA—C7B—H7BB	107.6	C19C—C18C—H18C	119.7
O1B—C8B—N1B	123.5 (3)	C17C—C18C—H18C	119.7
O1B—C8B—C7B	122.9 (3)	C18C—C19C—C14C	118.5 (3)
N1B—C8B—C7B	113.6 (2)	C18C—C19C—H19C	120.7
C10B—C9B—N1B	129.9 (3)	C14C—C19C—H19C	120.7
C10B—C9B—C13B	108.5 (2)		
			
C10A—N2A—N3A—C13A	−7.7 (3)	N1B—C9B—C10B—C11B	−5.7 (5)
C12A—N2A—N3A—C13A	−142.5 (2)	C13B—C9B—C10B—C11B	176.5 (3)
C10A—N2A—N3A—C14A	−165.7 (2)	N3B—N2B—C10B—C9B	6.1 (3)
C12A—N2A—N3A—C14A	59.5 (3)	C12B—N2B—C10B—C9B	140.2 (3)
C6A—C1A—C2A—C3A	0.3 (5)	N3B—N2B—C10B—C11B	−173.2 (2)
C7A—C1A—C2A—C3A	−177.4 (3)	C12B—N2B—C10B—C11B	−39.0 (4)
C1A—C2A—C3A—C4A	1.4 (5)	N2B—N3B—C13B—O2B	−172.6 (2)
C1A—C2A—C3A—Cl1A	−178.2 (3)	C14B—N3B—C13B—O2B	−16.1 (4)
C2A—C3A—C4A—C5A	−1.0 (5)	N2B—N3B—C13B—C9B	5.7 (3)
Cl1A—C3A—C4A—C5A	178.6 (3)	C14B—N3B—C13B—C9B	162.2 (2)
C2A—C3A—C4A—Cl2A	178.5 (3)	C10B—C9B—C13B—O2B	176.3 (3)
Cl1A—C3A—C4A—Cl2A	−1.9 (5)	N1B—C9B—C13B—O2B	−1.7 (4)
C3A—C4A—C5A—C6A	−0.9 (6)	C10B—C9B—C13B—N3B	−1.9 (3)
Cl2A—C4A—C5A—C6A	179.5 (3)	N1B—C9B—C13B—N3B	−179.9 (2)
C4A—C5A—C6A—C1A	2.6 (6)	C13B—N3B—C14B—C15B	−117.5 (3)
C2A—C1A—C6A—C5A	−2.2 (5)	N2B—N3B—C14B—C15B	36.4 (4)
C7A—C1A—C6A—C5A	175.4 (3)	C13B—N3B—C14B—C19B	61.1 (4)
C2A—C1A—C7A—C8A	−56.3 (4)	N2B—N3B—C14B—C19B	−145.0 (3)
C6A—C1A—C7A—C8A	126.1 (3)	C19B—C14B—C15B—C16B	−1.7 (5)
C9A—N1A—C8A—O1A	−5.1 (4)	N3B—C14B—C15B—C16B	176.8 (3)
C9A—N1A—C8A—C7A	176.8 (3)	C14B—C15B—C16B—C17B	0.0 (6)
C1A—C7A—C8A—O1A	26.4 (5)	C15B—C16B—C17B—C18B	1.6 (6)
C1A—C7A—C8A—N1A	−155.4 (3)	C16B—C17B—C18B—C19B	−1.6 (6)
C8A—N1A—C9A—C10A	59.5 (4)	C17B—C18B—C19B—C14B	−0.1 (5)
C8A—N1A—C9A—C13A	−123.7 (3)	C15B—C14B—C19B—C18B	1.8 (5)
N1A—C9A—C10A—N2A	175.2 (3)	N3B—C14B—C19B—C18B	−176.8 (3)
C13A—C9A—C10A—N2A	−2.0 (3)	C10C—N2C—N3C—C13C	6.7 (3)
N1A—C9A—C10A—C11A	−4.9 (5)	C12C—N2C—N3C—C13C	139.3 (2)
C13A—C9A—C10A—C11A	178.0 (3)	C10C—N2C—N3C—C14C	166.5 (2)
N3A—N2A—C10A—C9A	5.9 (3)	C12C—N2C—N3C—C14C	−60.9 (3)
C12A—N2A—C10A—C9A	137.2 (3)	C6C—C1C—C2C—C3C	−0.4 (5)
N3A—N2A—C10A—C11A	−174.1 (2)	C7C—C1C—C2C—C3C	−179.8 (3)
C12A—N2A—C10A—C11A	−42.7 (4)	C1C—C2C—C3C—C4C	0.2 (5)
N2A—N3A—C13A—O2A	−172.2 (2)	C1C—C2C—C3C—Cl1C	−179.1 (3)
C14A—N3A—C13A—O2A	−15.3 (4)	C2C—C3C—C4C—C5C	0.8 (5)
N2A—N3A—C13A—C9A	6.4 (3)	Cl1C—C3C—C4C—C5C	−179.9 (3)
C14A—N3A—C13A—C9A	163.3 (2)	C2C—C3C—C4C—Cl2C	179.5 (3)
C10A—C9A—C13A—O2A	175.8 (3)	Cl1C—C3C—C4C—Cl2C	−1.2 (4)
N1A—C9A—C13A—O2A	−1.6 (5)	C3C—C4C—C5C—C6C	−1.7 (5)
C10A—C9A—C13A—N3A	−2.8 (3)	Cl2C—C4C—C5C—C6C	179.6 (3)
N1A—C9A—C13A—N3A	179.8 (2)	C2C—C1C—C6C—C5C	−0.5 (5)
C13A—N3A—C14A—C15A	−122.7 (3)	C7C—C1C—C6C—C5C	178.9 (3)
N2A—N3A—C14A—C15A	31.7 (4)	C4C—C5C—C6C—C1C	1.5 (6)
C13A—N3A—C14A—C19A	56.1 (4)	C6C—C1C—C7C—C8C	117.2 (3)
N2A—N3A—C14A—C19A	−149.5 (3)	C2C—C1C—C7C—C8C	−63.4 (4)
C19A—C14A—C15A—C16A	0.1 (5)	C9C—N1C—C8C—O1C	8.7 (4)
N3A—C14A—C15A—C16A	178.9 (3)	C9C—N1C—C8C—C7C	−169.1 (3)
C14A—C15A—C16A—C17A	−0.9 (5)	C1C—C7C—C8C—O1C	26.7 (4)
C15A—C16A—C17A—C18A	1.4 (6)	C1C—C7C—C8C—N1C	−155.5 (3)
C16A—C17A—C18A—C19A	−1.0 (6)	C8C—N1C—C9C—C10C	−58.1 (4)
C15A—C14A—C19A—C18A	0.3 (5)	C8C—N1C—C9C—C13C	122.4 (3)
N3A—C14A—C19A—C18A	−178.6 (3)	N1C—C9C—C10C—N2C	−178.0 (2)
C17A—C18A—C19A—C14A	0.2 (5)	C13C—C9C—C10C—N2C	1.6 (3)
C10B—N2B—N3B—C13B	−7.4 (3)	N1C—C9C—C10C—C11C	1.5 (5)
C12B—N2B—N3B—C13B	−145.0 (2)	C13C—C9C—C10C—C11C	−178.9 (3)
C10B—N2B—N3B—C14B	−164.4 (2)	N3C—N2C—C10C—C9C	−5.0 (3)
C12B—N2B—N3B—C14B	58.0 (3)	C12C—N2C—C10C—C9C	−134.8 (3)
C6B—C1B—C2B—C3B	−0.5 (5)	N3C—N2C—C10C—C11C	175.4 (2)
C7B—C1B—C2B—C3B	−179.8 (3)	C12C—N2C—C10C—C11C	45.6 (4)
C1B—C2B—C3B—C4B	−0.2 (5)	N2C—N3C—C13C—O2C	173.1 (2)
C1B—C2B—C3B—Cl1B	177.6 (3)	C14C—N3C—C13C—O2C	15.1 (4)
C2B—C3B—C4B—C5B	0.0 (5)	N2C—N3C—C13C—C9C	−5.7 (3)
Cl1B—C3B—C4B—C5B	−177.8 (3)	C14C—N3C—C13C—C9C	−163.7 (3)
C2B—C3B—C4B—Cl2B	179.2 (3)	C10C—C9C—C13C—O2C	−176.2 (3)
Cl1B—C3B—C4B—Cl2B	1.3 (4)	N1C—C9C—C13C—O2C	3.4 (5)
C3B—C4B—C5B—C6B	0.9 (5)	C10C—C9C—C13C—N3C	2.5 (3)
Cl2B—C4B—C5B—C6B	−178.2 (3)	N1C—C9C—C13C—N3C	−177.9 (2)
C4B—C5B—C6B—C1B	−1.6 (6)	C13C—N3C—C14C—C15C	125.5 (3)
C2B—C1B—C6B—C5B	1.4 (5)	N2C—N3C—C14C—C15C	−30.7 (4)
C7B—C1B—C6B—C5B	−179.3 (3)	C13C—N3C—C14C—C19C	−54.1 (4)
C2B—C1B—C7B—C8B	60.7 (4)	N2C—N3C—C14C—C19C	149.7 (3)
C6B—C1B—C7B—C8B	−118.6 (3)	C19C—C14C—C15C—C16C	−0.8 (5)
C9B—N1B—C8B—O1B	−10.9 (5)	N3C—C14C—C15C—C16C	179.6 (3)
C9B—N1B—C8B—C7B	166.8 (3)	C14C—C15C—C16C—C17C	0.7 (6)
C1B—C7B—C8B—O1B	−29.8 (5)	C15C—C16C—C17C—C18C	0.1 (7)
C1B—C7B—C8B—N1B	152.5 (3)	C16C—C17C—C18C—C19C	−0.7 (7)
C8B—N1B—C9B—C10B	60.3 (4)	C17C—C18C—C19C—C14C	0.6 (5)
C8B—N1B—C9B—C13B	−122.2 (3)	C15C—C14C—C19C—C18C	0.2 (5)
N1B—C9B—C10B—N2B	175.1 (3)	N3C—C14C—C19C—C18C	179.8 (3)
C13B—C9B—C10B—N2B	−2.7 (3)		

### Hydrogen-bond geometry (Å, º)

**Table:**

*D*—H···*A*	*D*—H	H···*A*	*D*···*A*	*D*—H···*A*
N1*A*—H1*AA*···O2*A*^i^	0.88	1.98	2.820 (3)	159
N1*B*—H1*BA*···O2*C*	0.88	2.01	2.849 (3)	159
N1*C*—H1*CA*···O2*B*	0.88	1.96	2.795 (3)	158
C11*A*—H11*C*···O2*B*^ii^	0.98	2.39	3.344 (4)	163
C11*C*—H11*H*···O2*A*^iii^	0.98	2.45	3.377 (4)	158
C12*C*—H12*G*···O2*A*^iii^	0.98	2.44	3.186 (4)	133
C12*C*—H12*H*···O1*B*^iii^	0.98	2.51	3.178 (4)	125
C12*A*—H12*A*···O2*B*^ii^	0.98	2.50	3.273 (4)	136
C11*B*—H11*F*···O2*C*^iv^	0.98	2.42	3.364 (4)	163
C12*B*—H12*D*···O2*C*^iv^	0.98	2.46	3.282 (4)	142
C17*B*—H17*B*···Cl1*B*^v^	0.95	2.89	3.705 (4)	144

Symmetry codes: (i) −*x*+1, −*y*+1, −*z*+2; (ii) −*x*+1, *y*−1/2, −*z*+3/2; (iii) *x*, −*y*+3/2, *z*−1/2; (iv) −*x*+1, −*y*+1, −*z*+1; (v) *x*+1, *y*, *z*.

## References

[bb1] Agilent (2011). *CrysAlis PRO* and *CrysAlis RED* Agilent Technologies, Yarnton, England.

[bb2] Allen, F. H. (2002). *Acta Cryst.* B**58**, 380–388. 10.1107/s0108768102003890 12037359

[bb3] Bernstein, J., Davis, R. E., Shimoni, L. & Chang, N.-L. (1995). *Angew. Chem. Int. Ed. Engl.* **34**, 1555–1573.

[bb4] Butcher, R. J., Mahan, A., Nayak, P. S., Narayana, B. & Yathirajan, H. S. (2013*a*). *Acta Cryst.* E**69**, o39. 10.1107/S1600536812049628 PMC3588232 23476425

[bb5] Butcher, R. J., Mahan, A., Nayak, P. S., Narayana, B. & Yathirajan, H. S. (2013*b*). *Acta Cryst.* E**69**, o46–o47. 10.1107/S160053681204963X PMC3588313 23476430

[bb6] Clark, R. C. & Reid, J. S. (1995). *Acta Cryst.* A**51**, 887–897.

[bb7] Fun, H.-K., Quah, C. K., Narayana, B., Nayak, P. S. & Sarojini, B. K. (2011*a*). *Acta Cryst.* E**67**, o2926–o2927. 10.1107/S1600536811041110 PMC3247340 22219958

[bb8] Fun, H.-K., Quah, C. K., Narayana, B., Nayak, P. S. & Sarojini, B. K. (2011*b*). *Acta Cryst.* E**67**, o2941–o2942. 10.1107/S1600536811041468 PMC3247353 22219971

[bb9] Fun, H.-K., Quah, C. K., Nayak, P. S., Narayana, B. & Sarojini, B. K. (2012*a*). *Acta Cryst.* E**68**, o2677. 10.1107/S1600536812034605 PMC3435699 22969570

[bb10] Fun, H.-K., Shahani, T., Nayak, P. S., Narayana, B. & Sarojini, B. K. (2012*b*). *Acta Cryst.* E**68**, o519. 10.1107/S1600536812002383 PMC3275261 22347117

[bb11] Mijin, D. & Marinkovic, A. (2006). *Synth. Commun.* **36**, 193–198.

[bb12] Mijin, D. Z., Prascevic, M. & Petrovic, S. D. (2008). *J. Serb. Chem. Soc.* **73**, 945–950.

[bb13] Sheldrick, G. M. (2008). *Acta Cryst.* A**64**, 112–122. 10.1107/S0108767307043930 18156677

[bb14] Wu, W.-N., Cheng, F.-X., Yan, L. & Tang, N. (2008). *J. Coord. Chem.* **61**, 2207–2215.

[bb15] Wu, W.-N., Wang, Y., Zhang, A.-Y., Zhao, R.-Q. & Wang, Q.-F. (2010). *Acta Cryst.* E**66**, m288. 10.1107/S160053681000471X PMC2983540 21580233

